# Metabolomic analysis of the serum and urine of rats exposed to diazinon, dimethoate, and cypermethrin alone or in combination

**DOI:** 10.1186/s40360-023-00714-6

**Published:** 2024-01-02

**Authors:** Yu-Jie Liang, Ding-Xin Long, Shanshan Wang, Hui-Ping Wang, Yi-Jun Wu

**Affiliations:** 1 Laboratory of Molecular Toxicology, State Key Laboratory of Integrated Management of Pest Insects and Rodents, Institute of Zoology, Chinese Academy of Sciences, 100101 Beijing, P. R. China; 2 School of Rehabilitation Medicine, Jining Medical University, 272067 Jining, Shandong P. R. China; 3 School of Public Health, University of South China, 421001 Hengyang, Hunan P. R. China; 4 Institute of Quality Standard and Testing Technology for Agro-products, Key Laboratory of Agro-product Quality and Safety, Chinese Academy of Agricultural Sciences, Ministry of Agriculture, 100081 Beijing, P. R. China

**Keywords:** Multiple-pesticide exposure, Subacute toxicity, Organophosphate, Pyrethroid, Metabolomics analysis

## Abstract

**Background:**

Multiple pesticides are often used in combination for plant protection and public health. Therefore, it is important to analyze the physiological changes induced by multiple pesticides exposure. The objective of this study was to investigate the combined toxicity of the widely-used organophosphorus and pyrethroid pesticides diazinon, dimethoate, and cypermethrin.

**Methods:**

Male Wistar rats were administrated by gavage once daily with the three pesticides individual or in combination for consecutive 28 days. The metabolic components of serum and urine samples were detected by using ^1^H nuclear magnetic resonance (NMR)-based metabolomics method. Histopathological examination of liver and kidneys and serum biochemical determination were also carried out.

**Results:**

The results showed that after the 28-day subacute exposure, serum glutamic transaminase and albumin were significantly increased and blood urea nitrogen was significantly decreased in the rats exposed to the mixture of the pesticides compared with the control rats, suggesting that the co-exposure impaired liver and kidney function. Metabolomics analysis indicated that the indicators 14 metabolites were statistically significant altered in the rats after the exposure of the pesticides. The increase in 3-hydroxybutyric acid in urine or decrease of lactate and N-acetyl-L-cysteine in serum could be a potentially sensitive biomarker of the subchronic combined effects of the three insecticides. The reduction level of 2-oxoglutarate and creatinine in urine may be indicative of dysfunction of liver and kidneys.

**Conclusion:**

In summary, the exposure of rats to pesticides diazinon, dimethoate, and cypermethrin could cause disorder of lipid and amino acid metabolism, induction of oxidative stress, and dysfunction of liver and kidneys, which contributes to the understanding of combined toxic effects of the pesticides revealed by using the metabolomics analysis of the urine and serum profiles.

## Introduction

Organophosphorus pesticides and pyrethroid insecticides are widely used in agriculture and public health. Diazinon (DZN) and dimethoate (DIM) are the two representatives of organophosphorus pesticides; while cypermethrin (CYP) is the one of pyrethroid insecticides. CYP exerts its insecticidal effect mainly by inhibiting the voltage-sensitive sodium channels, and has been widely used to control a variety of pests in cotton, fruits and vegetables, etc. Although CYP has a high hydrophobicity and high stability in soil, it is easily transported to water through runoff and soil erosion [1]. DZN and DIM, two commonly-used organophosphorus pesticides, display neurotoxic effect by inhibiting cholinesterase [2]. The acute toxicity of these pesticides has been well documented [3–5]. Liver and kidney, which are the responsible organs for metabolism, are common targets of the toxicity of the pesticides [6, 7]. Exposure to DZN, DIM or CYP could induce hepatotoxicity and nephrotoxicity with oxidative stress [8–10].

As DZN, DIM or CYP was used in large quantities, their residues are often detected in soil, water, and air; they often coexist in the environmental media. In agriculture practice, it has become more and more common to use a mixture of pesticides to overcome resistance of pests to a single pesticide. Thus multiple-pesticide residues likely coexist in the same environment [11, 12]. The pesticides residues in environmental media leads to their accumulation in food chain. For example, multiple pesticides originating from plant protection treatments and pest control were often detected in beehive substrates [13]. Actually, the pesticides residues can be found in drinking water, food, and agricultural products.

Based on the different modes of action, the combination of CYP, DZN and/or DIM was generally thought to be more toxic than any single one of three chemicals. However, the combined effects of these chemicals remain largely unexplored although the three chemicals are used in combination in some commercial insecticides and the residues of the pesticides are found common in environmental medium.

However, combined use of pesticides are indeed toxic to humans, not only at high doses, causing acute toxicity, but even at low doses long-term exposure may lead to a range of health damages, including cancer and neurodegenerative diseases [14, 15], reproductive and developmental toxicity [16, 17] and respiratory effects [18]. In particular, exposure to multiple pesticides during the critical period of early development could induce lasting adverse effects. The combination of pesticides may cause different toxicity from the individual toxicity.

In recent years, metabolomics analysis techniques have provided important information on the toxicology of pesticides, especially multiple pesticides in combination. Metabolomics focuses on the endogenous metabolites or their changes over time before and after stimulation of biological systems by exogenous contaminants [19]. The overall changes in metabolites are the ultimate expression of gene expression as well as all enzyme activities in metabolic pathways, which directly reveals the physiological state of the organisms [20]; the effects of multiple pesticides on metabolic pathways can also be determined based on the pattern of metabolite changes before and after exposure. The use of metabolomics to study the toxicological effects of pesticides on experimental animals mouse and rat can help to predict the potential toxicological effects of pesticides on mammals and provide the mechanisms underlying of pesticide toxicity. The discovery of major metabolites can lead to the identification of potentially sensitive biomarkers in major target tissues following pesticide exposure. In addition, biomarker studies can provide a reference point for understanding the mechanisms of specific toxicity. Previously, we conducted metabolic toxicological studies of carbamates, organophosphates, pyrethroids and other pesticides [21–25]. The metabolomic abnormalities after the exposure of pesticides may suggest different toxic effects of pesticides on fatty acid and energy metabolism, antioxidant defense system, DNA structure, and liver and kidney function [26].

Nuclear magnetic resonance (NMR) spectroscopy has been proven to be an effective method to obtain high quality quantitative information of metabolic processes within whole organisms [27–30]. This technique allows simultaneous detection of hundreds of low-molecular weight species within a biological matrix and generates an endogenous metabolic profile that is altered characteristically in response to external stimuli. The pattern recognition technology, such as principal components analysis (PCA), can facilitate the visualization of inherent patterns in the data and characterization of novel biomarkers [19, 24, 31]. Metabolomics can be employed to solve a wide range of problems in the biomedical and clinical areas, especially in toxicology [32]. We have reported the changes in serum metabolic profiles from rats after exposure to chlorpyrifos, carbaryl, or a combination of these pesticides [26, 33].

In this study, we investigated the subacute toxicity of DZN, DIM, CYP, and their combination in rats by using integrated metabolomic approach coupled with biochemical methods to shed light on the toxicological mechanism of the pesticides. We tested the metabolic changes in urine and serum of rats after subacute exposure of DZN, DIM, and CYP (individual and in combination). We analyzed the changes in metabolic fingerprints to determine the toxic mode of action of the three pesticides and to identify potential biomarkers for the toxicity.

## Materials and methods

### Chemicals

Diazinon (DZN) (95% purity) was donated by Zhangjiagang Aihua Chemical Co. Ltd. (Jiangsu, China). Dimethoate (DIM) (97% purity) was obtained from Chongqing Pesticide & Chemical Industry (GROUP) Co. Ltd (Chongqing, China). Cypermethrin (CYP) (95% purity) was obtained from Wuxi Hemei Agrochemical Co. Ltd. (Jiangsu, China). Sodium pentobarbital were purchased from Sinopharm Chemical Reagent Co. Ltd (Shanghai, China). D_2_O and 2, 2, 3, 3-trimethysilylpropioinic-2,2,3,3-acide (TSP) were purchased from Sigma-Aldrich Chemical Company (St Louis, MO, USA). All other reagents were of analytical grade and obtained from commercial sources.

### Animals and treatments

Thirty male Wistar rats (6 weeks old, weighing 180–220 g) were obtained from Beijing Vital River Laboratory Animal Technology Co., Ltd (Beijing, China). After arrival, they were allowed to acclimatize for a week before the start of the experimentation. The animals were housed in groups under controlled conditions of temperature (25 °C), relative humidity (50%), and photoperiod (12-h light/12-h dark cycle). All animals were allowed freely available to food and water.

The animals were weighed and then randomly divided into the following five experimental groups (the mean initial body weight of each group was not significantly different): CTL(vehicle, control), DZN (diazinon, 5 mg/kg), DIM (dimethoate, 6 mg/kg), CYP (cypermethrin, 10 mg/kg), and MIX (diazinon, 5 mg/kg plus dimethoate, 6 mg/kg plus cypermethrin, 10 mg/kg) with 6 rats in each group. Doses of the pesticides were chosen to below those expected to cause overt toxic signs in rat. Thus 1/50 of half-lethal doses (LD_50_) was used as the administration dose, for each pesticide based on our study in rats [34]. As reported in the literature, acute oral LD_50_ of DZN, DIM, and CYP were 250, 300, and 500 mg/kg/day, respectively [35–37]. All pesticides were suspended in corn oil at a volume dose of 1 ml/kg of rat body weight before being administered. The animals were administered through oral gavage once daily for 28 consecutive days. The control rats were gavaged with the corresponding volume of the carrier oil. All procedures involving the use of live animals as described in this study were approved by the Animal Ethics Committee of Institute of Zoology, Chinese Academy of Sciences. All animals were cared for in accordance with the National Institutes of Health (NIH) guidelines for the care and use of laboratory animals. The study was also conducted according to ARRIVE guidelines [38].

### Sample collection and preparation

After the 28-day oral administration, 24-hour urine samples were collected using a metabolic cage with 1% NaN_3_ solution added as a preservative. The collected urine samples were centrifuged at 3,000 × g for 10 min to remove debris, and then 400 μl of the supernatant was transferred to polypropylene tubes and stored at -80 °C for NMR detection. At the end of the experiment, the rats were anesthetized by intraperitoneal injection of sodium pentobarbital (40 mg/kg body weight), after the collection of 24-hour urine. Then the animals were decapitated and the different tissues were quickly dissected and blood samples were collected when the death of the animals was confirmed (the heart was not beating). The serum samples were prepared and stored at − 80 °C for ^1^H NMR analysis and biochemical analysis.

### Histopathological analysis

For histopathological analysis, the kidney and liver tissues of the rats were dissected immediately at the end of the 28-day exposure to the pesticides, and then were fixed in a solution of 10% formalin. The fixed specimens were dehydrated in a graded ethanol series (80%, 95%, and 100%), and then embedded in paraffin. The paraffin blocks were sliced into 4-μm thick sections using a microtome (Microm HM 340E, Thermo Fisher Scientific, USA). The slides were deparaffinized with three changes of xylene and rehydrated through a graded ethanol-water series. Standard hematoxylin and eosin (H&E) staining procedure was used to stain the tissue sections. The histological changes in the kidney and liver tissue sections were examined in a microscope (Olympus, Tokyo, Japan).

### Biochemical analysis

The biochemistry of serum samples were detected on a Biochemical Autoanalyzer (Type 7170, Hitachi, Japan) by following the chemical parameters: serum glutamic-oxaloacetic transaminase (SGOT), serum glutamic-pyruvic transaminase (SGPT), blood glucose (GLU), blood urea nitrogen (BUN), alkaline phosphatase (ALP), albumin (ALB), and creatinine (CRE).

### Preparation of the ^1^H NMR samples

Prior to the analysis of NMR, the serum and urine samples were thawed to room temperature, centrifuged at 13,000 × g at 4 °C for 10 min and the supernatants were collected. Then, 400 μl of the supernatant was mixed with 200 μl of phosphate buffer solution (PBS, pH 7.4) to eliminate the deviation of the chemical shift of the peaks due to the pH change. After vortexed for 60 s, the mixture was centrifuged at 3.500 × g for 10 min to remove any precipitates. Then, 450 μl of the supernatant was transferred into a 5-mm NMR sample tube. Then 50 μl of D_2_O solution containing 1 mg/ml TSP was added. The D_2_O was used to lock the field, and TSP was used as the reference for chemical shift (δ 0.0).

### NMR data acquisition and analysis

The methods for NMR data acquisition and analysis were referenced to the literature [28, 39]. The urine sample data were acquired on a Bruker Avance-600 Spectrometer with a pre-saturation method to suppress the water peak, 64 K data points, 5 S sampling delay, and 90 °C deflection angle for the RF pulse. The NMR data were acquired by Fourier transformation following apodization and zero filling of the free induction decay (FID). Then ^1^H chemical shifts are reference to TSP at 0 ppm. After the re-phase correction and baseline correction, the water peak and the shift 4.2 ~ 6.2 ppm where the urea-like active hydrogen caused by cross relaxation were removed, and the remaining 196 spectral integrals were segmented from 0 ~ 10 at 0.04 ppm intervals to extract the integral value data. The ^1^H NMR spectra of all serum samples were obtained at 298 K. A one-dimensional Carr-Purcell-Meiboom-Gill (CPMG) [recycle delay-90°(τ-180°-τ)n-acquisition] pulse sequence with water suppression was used to selectively obtain signals with low molecular weight. To eliminate the influence of the methanol and water peaks, the 3.36–3.37 ppm and 4.7–5.2 ppm areas were excluded. After normalizing the integration area, these data were used for principal component analysis in pattern recognition.

### Multivariate analysis

The obtained data matrix was subjected to multivariate chemometric analysis in SIMCA-P (version 12.0; Umetrics, Umea, Sweden). A principal component analysis (PCA) and a subsequent orthogonal partial least squares discriminant analysis (OPLS-DA) were included to distinguish differences between treatment groups. The results were presented in the form of scores plot and corresponding loadings plot.

In the scores plot, the first principal component includes most of the variables, and the first and second principal components provide a two-dimensional data representing the relevant variables of each sample [40, 41], reflecting the distribution relationship between the samples, from which the differences between the groups of samples can be derived; while the corresponding factor loadings plot reflects the contribution of each integral segment to the scores of the principal components of the samples [41], The quality control of each model was determined by the goodness-of-fit parameter and the predictability parameter as well as the proportion of total variance predicted by a component. Biomarkers were then identified using loading plots for each bin in the model and the variable importance in projection (VIP) scores [42].

## Results

### Serum biochemistry

The changes of some key serum parameters were found by biochemical analysis. Compared with the control rats, serum of the exposed rats in exhibited obvious disturbance (Table 1). The activity of SGPT from the rats exposed to diazinon or dimethoate was significantly lower than that from the control rats (*P* < 0.05). The levels of SGOT, ALP, and ALB of the rats exposed to the combination of the three pesticides were significantly increased, while BUN in the rats exposed simultaneously to the three pesticides and the rats to CYP alone was significantly decreased compared with that from the control rats, indicating dysfunction of liver and kidneys. It is known that SGOT is a key enzyme that is related to liver function In this study, the increase of SGOT activity and the decrease of GLU suggested that the mixture of CYP, DIM, and DZN or CYP alone could cause liver damage.

### Histopathology

**Fig. 1:**
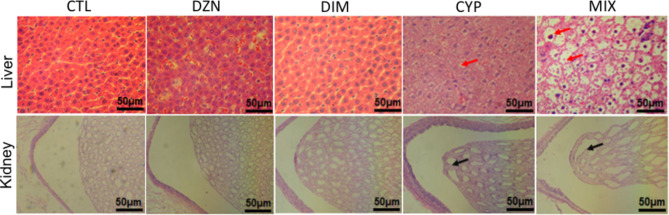
Effect of pesticides diazinon (DZN), dimethoate (DIM), and cypermethrin (CYP) alone or in combination on the liver and kidneys of rats. The tissue sections of liver and kidneys of the rats were examined under a microscope. Compared with the control rats showing normal structure of liver (upper panel) and kidney tissues (bottom panel), the rats exposed to DZN (250 mg/kg body weight/day), DIM (300 mg/kg body weight/day), and CYP (500 mg/kg body weight/day) individual or in combination for 28 consecutive days exhibited swelling degeneration of hepatocytes in liver tissue (the red arrows indicate the hepatocyte necrosis) and abnormal glomerular capillary wall in kidney tissue (the black arrows indicate the glomerular lesions). Scale bar: 50 μm. Abbreviation: CTL, control; MIX, the mixture of DZN, DIM, and CYP

Histopathological examination displayed representative images of hematoxylin and eosin (H&E) staining of the liver and kidneys of rats. There are cloudy and swollen hepatocytes and pathological changes such as vacuolar degeneration in the liver of the rats exposed to the combination of the three pesticides (Fig. 1, upper panel). Significant pathological changes such as nuclear concentration was also observed in the liver tissue of CYP-exposed rats. The histological changes of liver from the rats exposed to multiple pesticides was more obvious.

The microscopic photos of kidney sections showed prominent glomerular lesions in the renal parenchyma of the rat exposed to CYP alone and combined with DIM and DZN (Fig. 1, bottom panel). These results indicated that the exposure of combination of three pesticides caused obvious manifestations of renal tissue damage.

**Table 1:** Effect of pesticides on serum biochemical parameters of rats (mean ± SD)

Parameters	Control	Diazinon	Dimethoate	Cypermethrin	Combination
SGOT (U/L)	156.5 ± 8.2	228.0 ± 20.4	206.5 ± 14.3	229.0 ± 21.8	255.2 ± 96.8^*^
SGPT (U/L)	62.0 ± 5.4	45.7 ± 3.9^*^	47.2 ± 6.5^*^	52.2 ± 5.5	58.5 ± 7.2
GLU (mM)	5.4 ± 0.1	5.7 ± 0.6	5.5 ± 0.2	4.4 ± 0.9^*^	5.0 ± 0.5
BUN (mM)	7.3 ± 1.4	7.5 ± 0.9	6.5 ± 0.7	5.6 ± 0.5^*^	4.8 ± 0.5^*^
ALP (U/L)	132.0 ± 16.6	157.5 ± 4.9	150.7 ± 9.8	137.2 ± 12.1	179.7 ± 46.7^*^
ALB (g/L)	33.0 ± 0.2	33.5 ± 0.9	32.8 ± 0.5	34.8 ± 1.9	35.7 ± 2.2
CRE (μM)	51.0 ± 4.2	55.5 ± 3.5	48.2 ± 6.4	39.5 ± 9.6	47.5 ± 7.5

Notes: * *P* < 0.05, compared with the control (n = 6 for each group). Abbreviations: SGOT, serum glutamic-oxaloacetic transaminase; SGPT, serum glutamic-pyruvic transaminase; GLU, glucose; BUN, blood urea nitrogen; ALP, alkaline phosphatase; ALB, albumin; CRE, creatinine

### Metabolic differences in urine of the exposed rats

**Fig. 2:**
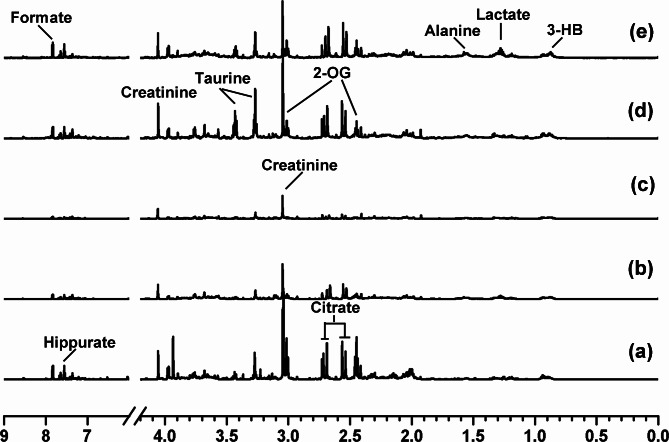
The representative spectrum of 600 MHz ^1^H NMR (0.5 ~ 4.5) spectra of urinary samples from the rats exposed to vehicle (**a**), diazinon (**b**), dimethoate (**c**), cypermethrin (**d**), or mixture of the three pesticides (**e**) for 28 consecutive days. Abbreviations: 2-OG, 2-oxoglutarate; 3-HB, 3-hydroxybutyric acid

**Fig. 3:**
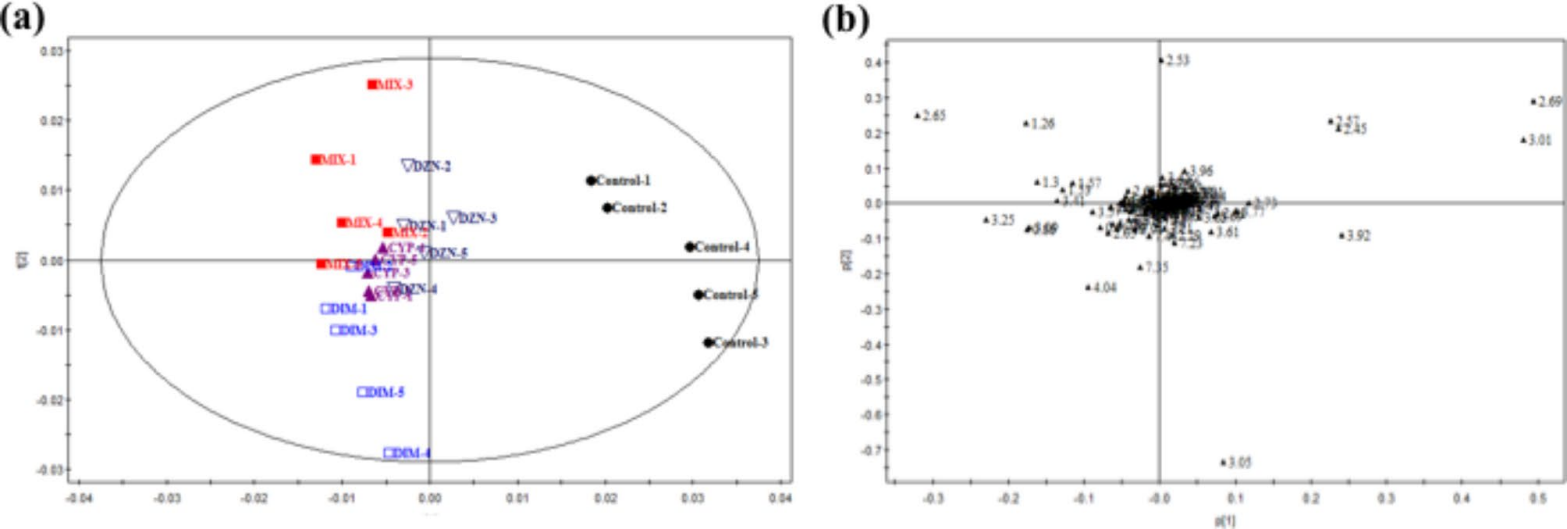
Partial least squares discriminate analysis (PLS-DA) from ^1^H NMR spectra of urine samples of the rats. The animals were exposed to three pesticides diazinon, dimethoate, and cypermethrin alone or in combination for 28 consecutive days, and then the 24 h-urine samples were collected and analyzed by an NMR spectrometer. Scores plot (**a**) and the loadings plot (**b**) for these models were used to distinguish which metabolites were the most significant in terms of describing this separation. Abbreviations: CYP, cypermethrin; DIM, dimethoate; DZN, diazinon; MIX, mixture of CYP, DIM, and DZN

The ^1^H NMR spectra of the urine samples obtained from the rats shows the average signal of the metabolites. As shown in Fig. 2, multiple species of metabolites were identified in the urine samples, including lipids, glucose, amino acids, and organic acids. Some metabolites like hippurate, citrate, and creatinine decreased in the DZN- or DIM-treated rats (Fig. 2b and c). In addition, the level of creatinine, taurine and 2-OG were increased in the CYP-exposed rats (Fig. 2d), while the main metabolites of urinary spectra consisted of amino acids were increased in the multiple-pesticide-exposed rats (Fig. 2e). Since this ^1^H-NMR spectra were obtained separately from a randomly selected rat, statistical analysis of the spectra of the endogenous metabolites needs to be done by multivariate analysis. PLS-DA was used to analyze the NMR data of the samples to determine overall metabolic trends and to identify possible metabolites. NMR spectral data from each group were therefore analyzed by PLS-DA to determine the metabolic profile affected by pesticide exposure. PLS-DA score plots showed a clear separation between the various groups (Fig. 3a). Five distinct sample groups were observed in the PLS-DA score plot. By analyzing the corresponding load scores (Fig. 3b) and raw NMR spectra for the various pesticide-exposed rats and control rats, it shows significant changes in urine composition due to the exposure of organophosphorus pesticides DZN and DIM. These metabolites include elevated lactic acid and acetic acid. And also, these metabolites such as citric acid and 2-ketoglutarate are all elevated to varying levels. As shown in the loading plots of Fig. 3b, more metabolites were increased in the combined pesticides, such as peaks in the positive direction of 3-hydroxybutyric acid (3-HB), which were increased more than in the control rats, suggesting abnormalities in energy metabolism in tricarboxylic acid cycle (TCA). Trimethyl phosphine oxide was also significantly elevated in CYP-treated rats compared with that in the control rats. The above results suggested that individual and combined pesticides of DZN, DIM, and CYP could effectively regulate the metabolic networks associated with some of these metabolites that are involved in energy metabolism.

### Metabolic changes in serum of rats exposed to multiple pesticides

**Fig. 4:**
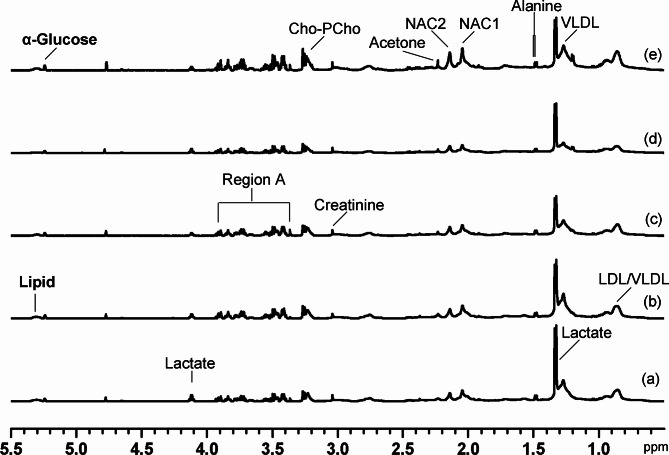
Typical CPMG ^1^H NMR spectra of serum from the rats exposed to vehicle (**a**), diazinon (**b**), dimethoate (**c**), cypermethrin (**d**), or mixture of the three pesticides (**e**) for 28 consecutive days. Abbreviations: Cho-PCho, choline-phosphocholine; NAC, N-acetyl-L-cysteine; LDL, low density lipoprotein; VLDL, very low density lipoprotein

**Fig. 5:**
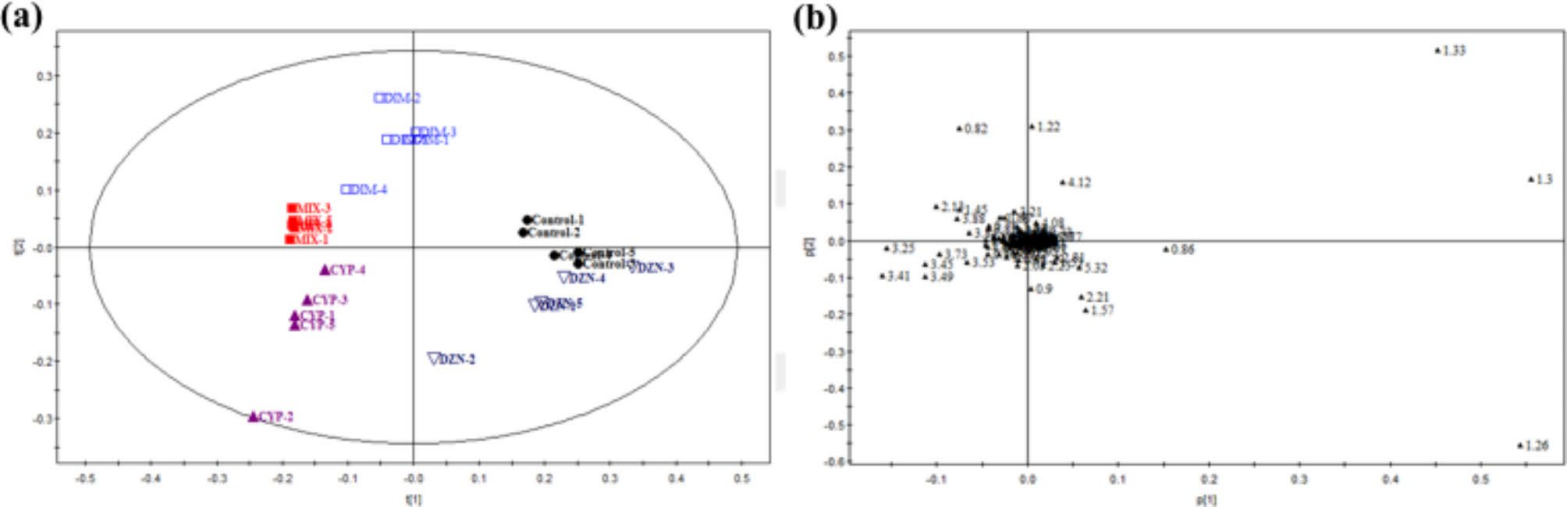
Partial least squares discriminate analysis (PLS-DA) from ^1^H NMR spectra of serum samples of rats. The animals were exposed to three pesticides diazinon, dimethoate, and cypermethrin alone or in combination for 28 consecutive days, and then the blood samples were collected and the serum was prepared and analyzed by an NMR spectrometer. Scores plot (**a**) and the loadings plot (**b**) for these models were used to distinguish which metabolites were the most significant in terms of describing this separation. Abbreviations: CYP, cypermethrin; DIM, dimethoate; DZN, diazinon; MIX, mixture of CYP, DIM, and DZN

Typical ^1^H NMR spectra of control rats serum and insecticide-treated rats are shown in Fig. 4a. We identified a range of serum metabolites including energy metabolism (lactate, creatinine, and glucose), lipid metabolism (low density lipoprotein/very low density lipoprotein, LDL/VLDL), amino acid metabolism (alanine), methylamine metabolism (choline) and ketone body metabolism (acetone). Although the level of the individual exposure of the pesticide DZN, DIM, or CYP did not show obvious perturbation of the main metabolites (Fig. 4b, c, and d), the metabolites including N-acetyl-L-cysteine (NAC), choline-phosphocholine (Cho-PCho), and alanine in serum from the rats exposed simultaneously to DZN, DIM, and CYP increased significantly (Fig. 4e). Again, we used PLS-DA to examine the possible changes in metabolic patterns between groups and to identify the key metabolites that contribute to changes in metabolic patterns (Fig. 5). PLS-DA allows a clear distinction between these five groups based on the serum metabolome (Fig. 5a). A series of metabolites were identified based on their corresponding VIP plots (Fig. 5b), which showed that enhanced ketone body signals were present in the serum ^1^H NMR spectra of rats in almost all of the combined groups after 28 days of administration.

The position of treatment points on each axis in scores plot could be influenced by variables on the same axis in a loading plot. In the loading plot, the isoleucine 1.26 (m) and lactate 1.33 (d) were located at the upper right corner of the quadrants, which corresponds to the upper right corner of the plot. This could indicate that the control group at the upper right corner has high concentration of isoleucine 1.26 (m) and lactate1.33 (d). Whereas, taurine 3.26, 3.42 (d) and NAC 2.13 (s) were located at the upper right in a given axis of the loading plot, which indicates the increased concentration in the same axis in the scores plot.

### Identification of dominant metabolites

In addition, these metabolites were analyzed and relatively quantitated. Table 2 shows the detailed correlations of serum and urine metabolites with each treatment group. We found a significant positive correlation for 3-HB in urine, and NAC and lactate in serum of the rats treated with the multiple pesticides. The concentration changes of 3-HB and lactate may reveal disturbance of energy metabolism. NAC is the acetylated derivative of the amino acid L-cysteine and could be as a potent antioxidant substance. As illustrated from Table 2, the metabolites 2-OG and creatinine were down-regulated in urine of the rats exposed to DIM or the three pesticides in combination, but citrate, 2-OG, and creatinine decreased only in the DIM-exposed rats correlating towards statistical significance.

**Table 2:** Summary of significantly changed urinary and serum metabolites from the rats treated with pesticides

Major metabolites		^1^H Chemical shifts (ppm)	DZN	DIM	CYP	MIX
Urine	Citrate	2.57, 2.69 (d)	↓*	↓*	↓	-
	2-OG	2.45, 3.01 (d)	-/↓	↓*	↓	↓
	Creatinine	3.05, 3.92 (d)	-/↓	↓*	-	↓
	3-HB	1.18 (d)	-/↓	-	↑	↑*
Serum	Isoleucine	1.26 (m)	-	↓	↓	↓
	Lactate	1.33, 4.12 (d)	-	↓	↓	↓*
	Taurine	3.25, 3.41 (d)	-/↓	↓	↑	↑
	NAC	2.13 (s)	-/↓	↑	↑	↑*

**Note**: s, singlet; d, doublet; t, triplet; m, multiplet. Changes are relative to control samples: “-”, no change; “↓”, decrease; “↑”, increase. The integral values for each segmented region of chemical shift were represented for each metabolite resonance. One-way ANOVA was used to assess for statistical significance of the integral values of assigned spectral peaks among the groups. * *p* < 0.05, compared with the control. Abbreviations: 2-OG, 2-oxoglutarate; 3-HB, 3-hydroxybutyric acid; NAC, N-acetyl-L-cysteine; CYP, cypermethrin; DIM, dimethoate; DZN, diazinon; MIX, mixture of CYP, DIM, and DZN.

## Discussion

Due to the application of various pesticides in combination, concerns have been raised over the last few decades about their potential risks to the environment and human health. While our previous work assessed the effects of single or two kinds of mixed pesticides on the toxicology of animal models, this paper provides an in-depth discussion of the toxicology of three combined pesticides.

In the present work, we performed ^1^H NMR-based metabolic analysis combined with histopathological and clinical biochemical assays to assess the toxic effects following the combination of three pesticides, as well as their potential molecular mechanisms. Our results showed the increased urinary levels of 3-HB in the composite group, which suggested that the simultaneous introduction of the three pesticides may have led to abnormalities in the metabolism, resulting in the accumulation of ketone bodies beyond the oxidative capacity of the peripheral tissues and thus affecting normal liver physiology. 3-HB is mainly regarded as a substrate for the synthesis of polyhydroxybutyric acid, which is a reserve substance. In animals, 3-HB is not only an intermediate metabolite but also an important regulatory molecule that can affect gene expression, lipid metabolism, neuronal function and overall metabolic rate [43]. The tricarboxylic acid (TCA) cycle intermediate 2-oxoglutarate (2-OG) is an essential substrate for a series of oxidation reactions. Decreased level of 2-OG indicates an abnormality in the cycle. 2-OG is involved in biological processes, including oxidation catalyzed by 2-OG oxygenase, which is a co-substrate [44]. The reduction level of 2-OG and creatinine in urine may be indicative of hepatotoxicity and nephrotoxicity. As shown in the corresponding loading plots of serum metabolism, some of the metabolic intermediates were found to be increased in both individual and combined pesticides, with citric acid and 2-ketoglutaric acid enhanced significantly in the combined pesticide exposed rats, indicating an abnormal energy metabolism in TCA cycle.

A study on the efficacy of DIM and CYP showed that CYP works immediately after application and remains effective for a long period of time, whereas DIM has a lag period before reaching maximum efficacy in a short period of time. This combination was usually found in the formulations of most commercial pesticides. The results of our study suggest that the coexistence of multiple pesticides may enhance toxicity to organisms. However, little research has been done on whether the effects of multiple pesticide combinations are additive or synergistic. Our metabolomics data in this study only indicate that the effects of combined toxicity are stronger. In addition, we found that patterns in changes of the main metabolites such as creatinine, taurine, and 2-OG were very similar between the CYP-treated rats and the MIX-treated rats. And we also noticed that the levels of these metabolites in the CYP-exposed rats were different from those in DZN- or DIM-exposed rats. These results suggested that CYP may contribute more to the combined toxicity of the three pesticides.

Traditional evaluation methods have limited observation indicators, low evaluation efficiency and insufficient sensitivity to meet the current evaluation needs, whereas metabolomics, as a novel research tool with the advantages of high sensitivity, high evaluation efficiency and comprehensive evaluation, has been widely used in environmental toxicology studies of pesticides. Compared with other analysis methods, metabolomics can completely reflect the changes in the types and relative contents of metabolites in organisms with pesticide exposure, and can be used to find the metabolites of pesticides in organisms as a whole or biomarkers after pesticide exposure, and also to explore the toxicological effects of pesticides and elucidate the toxicity mechanisms. The application of pesticide biomarkers in pesticide risk assessment and management should be further enhanced in the future to better serve as the environmental assessment of pesticides and health risk assessment of human exposure. At the same time, the current detection tools are still far from the goal of detecting all metabolites of an organism, and there is a need to develop new detection tools to gradually expand the scope of detection, so that metabolomics technology can be better applied to the environmental toxicology of pesticides. In addition, metabolites are often end products, and these end products may be influenced by many metabolic pathways. Therefore, the endogenous metabolites that are altered by metabolomics usually cannot be focused on a single identified metabolic pathway, and the combination of metabolomics with transcriptomics, proteomics and other -mics techniques is an important tool for future in-depth investigation into the mechanisms of pesticide environmental toxicology.

## Conclusion

Exposure to a combination of three pesticides diazinon, dimethoate, and cypermethrin may cause greater hepatotoxicity and nephrotoxicity. The reduction of 2-OG and creatinine in urine may be a potentially sensitive biomarker. These findings suggest that one of the important mechanisms of pesticide mixtures is by inhibiting lipid and amino acid metabolism and inducing oxidative stress, which provides essential information for metabolomics in evaluating the combined toxicity of pesticides.

## Data Availability

The datasets used and analyzed during the current study are available from the corresponding author on reasonable request. All data generated or analyzed during this study are included in this published article.
